# Investigating the joint application of negative pressure wound treatment and tissue therapy for chronic wounds in patients with diabetes

**DOI:** 10.25122/jml-2023-0020

**Published:** 2023-07

**Authors:** Yulia Ivanova, Svitlana Gramatiuk, Igor Kryvoruchko, Mykhailo Tymchenko, Kyrylo Goltsev, Karine Sargsyan

**Affiliations:** 1 Institute of Bio-Stem Cell Rehabilitation, Ukraine Association of Biobank, Kharkiv, Ukraine; 2 Surgery Department, Kharkiv National Medical University, Kharkiv, Ukraine; 3 International Biobanking and Education, Medical University of Graz, Graz, Austria; 4 Zaytsev V.T. Institute of General and Urgent Surgery, National Academy of Medical Sciences of Ukraine, Kharkiv, Ukraine

**Keywords:** diabetic foot syndrome, negative pressure wound treatment, human amniotic membrane, ABI: ankle-brachial, CFU: colony-forming units, GLASS: The Global Limb Anatomic Staging System, NPWT: Negative Pressure Wound Treatment, TpO2: tissue oxygenation, VAC: Vacuum-assisted closure

## Abstract

This study aimed to investigate the effectiveness of combined negative pressure wound therapy (NPWT) and human amniotic membrane in patients with chronic wounds associated with diabetes. A total of five patients with type 2 diabetes, including ischemic and mixed forms of diabetic foot syndrome, presenting with ischemic wounds of the lower extremities were included in this study. Patients with uncorrected limb ischemia were excluded. The treatment protocol included diabetes compensation (treatment with fractional insulin therapy), anticoagulant, metabolic therapy and angiotropic therapy, physical treatment methods, osteoporosis therapy with calcium preparations, and wound-specific interventions. The primary treatment approach involved the application of a vacuum bandage to the transplanted human amniotic membrane, which improved the adaptation of the flap to the wound surface, allowed the removal of excess wound exudate, and stimulated angiogenesis and reparative properties. The combined approach of NPWT and biotherapy was a safe and effective cure for diabetic wounds, promoting faster wound healing, reducing the need for autodermoplasty, and possibly reducing the necessity for high-level amputations.

## INTRODUCTION

The general prevalence of diabetes was evaluated at 9.3% (463 million people) in 2019 and is projected to rise to 10.2% (578 million) by 2030 and 10.9% (700 million) by 2045 [1, 2]. This chronic condition exerts metabolic changes that affect various organs and systems, leading to numerous complications, including severe late complications such as myocardial, renal, retinal, and lower extremity vascular disorders [3]. Among these, diabetic foot syndrome (DSS) holds one of the leading positions [3, 4]. Patients with diabetes are 20 times more likely to develop purulent-necrotic lesions in their feet than those without diabetes [3, 5].

The pathogenesis of the neuropathic form of diabetic foot syndrome is based on diabetic polyneuropathy, osteoarthropathy, and microangiopathy. The specified pathomorphological changes in almost all anatomical structures of the foot lead to the development of its deformation, hyperkeratosis, and the appearance of trophic ulcers. Angiopathy is one of the key links in the pathogenesis of the mixed form of diabetic foot syndrome. Macroangiopathy, which refers to the damage of large blood vessels, is a common and significant contributor to limb amputation in diabetic patients. The clinical course of the mixed form of diabetic foot syndrome is characterized by the interplay of neuropathy, angiopathy, and infection, each exacerbating the effects of the other. Moreover, angiopathy weakens the mechanisms of demarcation of the purulent-necrotic process and explains the very high risk and frequency of amputation in this category of patients [6, 7].

Treatment of purulent-necrotic processes in patients with diabetes mellitus is difficult given that the course of the wound process in patients with diabetes is characterized by the duration of the first phase and a reduced ability of tissues for reparative processes [8, 9]. The comprehensive surgical treatment of purulent-necrotic lesions in diabetes involves several essential components. These include diabetes management and control, immobilization or unloading of the affected limb, local therapy of ulcerative-necrotic lesions using modern dressings, targeted systemic antibiotic therapy, stopping the phenomena of critical ischemia, surgical treatment of a purulent-necrotic focus based on the principle of active surgical treatment. The active surgical approach entails wide access to the infection center, full revision and excision of all non-viable tissues, and plastic closure of wound and ulcer defects [6, 10].

Modern surgery has witnessed the emergence of diverse tools and techniques for the local treatment of ulcerative-necrotic lesions in patients with diabetic foot syndrome. Negative Pressure Wound Treatment (NPWT) and Vacuum-assisted Closure (VAC therapy) have gained significant recognition in the management of chronic wounds [11]. It has been widely used in various countries and, in recent years, also in Ukraine [12-14]. Vacuum therapy is one of the most widely used methods of treating various lesions in patients with diabetes. When applied in accordance with indications and proper methodology, its effectiveness has been observed across various stages of the wound-healing process [15, 16]. Extensive evidence from numerous randomized clinical trials supports the effectiveness and safety of NPWT in the comprehensive treatment of wound and ulcer defects in patients with diabetes. It has been proven that almost all infected and non-infected wounds can be treated with NPWT [17].

At the same time, the development of biotechnology has opened new possibilities for addressing the challenges associated with wound healing, including for patients with diabetes. Thus, platelet-rich alto plasma is increasingly used for treating trophic ulcers and chronic wounds - a source of growth factors that attract progenitor cells to the injured area and stimulate the proliferative activity of leukocytes in the wound. Israeli scientists proposed the CureXcell technology - local cytokine therapy by applying a suspension of donor leukocytes to the wound. Cellular technologies have also shown promise in wound healing, including methods such as culturing keratinocytes on collagen gel, growing epidermal cells on fibroblast cultures to create a “living equivalent of skin,” and utilizing combined substrates like Apligraf (allofibroblasts on a collagen gel matrix) along with cultured keratinocytes. Two-layer “artificial skin” composed of silicone film and the biodegradable membrane made of collagen and chondroitin-6-sulfate (Integra), as well as “cultured epidermal autografts” (Epicell, Epidex, Myskin), suspension of cultured autogenous keratinocytes (ReCell), fibroblasts on an organic silicon basis, products based on the cell-free allogeneic dermis (AlloDerm) and others have also shown promise [18-25].

Despite the availability of these advanced therapeutic approaches, there is currently a lack of studies exploring the combination of negative pressure therapy with tissue therapy. Therefore, this study aimed to investigate the effectiveness of combined NPWT and human amniotic membrane therapy in patients with chronic wounds associated with diabetes.

## MATERIAL AND METHODS

### Study participants

A total of five patients, both male and female, aged between 52 and 66, with wounds associated with type 2 diabetes (including ischemic and mixed forms of diabetic foot syndrome) were included in the study. Patients were treated and observed at the Department of Pathology of Main Vessels at the Institute of General and Urgent Surgery, named after Zaytsev V. T. of the National Academy of Medical Sciences of Ukraine in 2021–2022. The inclusion criteria for the study were the presence of type 2 diabetes and the presence of wounds. Patients with uncorrected limb ischemia (ankle-brachial index (ABI): 0.5; tissue oxygenation (TpO2): 25 mm Hg) were excluded from the study.

### Treatment protocol

Patients were treated according to the following scheme: compensation for diabetes (transfer to fractional insulin therapy), metabolic therapy, anticoagulant and angiotropic therapy, physical methods of treatment, and therapy aimed at treating osteoporosis (calcium preparations).

### Diagnostic procedures

The diagnosis of diabetic foot syndrome was established using a standard algorithm, which involved a detailed patient history and clinical and laboratory examination. The duration of the disease, intermittent lameness, pain at rest, presence and nature of soft tissue damage, pulse on main arteries, analysis of laboratory research methods, and history of concomitant diseases were considered. Non-invasive examinations were conducted to further assess the condition. This included determining the regional systolic pressure index on the foot arteries using a portable Super Dopplex ultrasound machine (China), Doppler ultrasound using the Hitachi EUB 7500 device (Japan) equipped with a linear sensor (L 5-10 MHz), and measurement of transcutaneous oxygen tension (TpO2) in the foot tissues using the TSM 400 device manufactured by Radiometer Copenhagen (Denmark). Invasive studies included angiography according to Seldinger, using the Philips Integris Allura (Holland) device. In addition, X-rays of the bones of the foot and lower leg were performed when necessary. The degree of ischemia was assessed according to the GLASS (2014) system.

### Endovascular interventions

Endovascular interventions were performed in four patients. The main procedure was percutaneous balloon angioplasty. After restoring the main blood flow in two patients, amputations of foot segments were performed.

### Wound treatment

We developed a method for treating wounds in patients with diabetes that involved two stages. In the first stage, NPWT sessions were performed to absorb wound discharge, stimulate microcirculation (in alternating mode), protect the wound defect from secondary infection by hospital strains of bacteria, and stimulate granulation growth. The level of negative pressure was adjusted below standard values in three patients with diabetic foot syndrome to prevent the appearance or progression of ischemic changes.

The effectiveness of NPWT was evaluated after the first bandage change, typically 2-3 days after the initiation of therapy. Positive dynamics, such as a reduction in edema, tissue infiltration, and the appearance of granulations, indicated the continuation of vacuum therapy. Once the wound bottom was filled with granulation tissue and the contamination level reduced below the critical threshold (≤10^3^ CFU/ml), the second stage of treatment was initiated. This involved covering the wound surface first with a human amniotic membrane, followed by a non-adhesive coating and a sorption sponge, with continuous monitoring of the NPWT system to select the optimal mode.

To temporarily close wounds, standard dressing kits for negative pressure wound therapy, KCI VAC Granufoam (KCI Manufacturing Unlimited Company, USA), and HEACO dressing kit DK10 (HEACO Medical Technologies, Great Britain), were used. The size and configuration of both the amniotic membrane and the open cell foam were selected depending on the size and configuration of the wound defect in such a way that the covering and the open cell foam completely filled not only the sac but also the pocket in the subcutaneous tissue. At the same time, in the case of pockets, the thickness of the open cell foam was reduced for more effective wound healing. A HEACO NP32S (HEACO Medical Technologies, Great Britain) was used to create negative pressure. An intermittent mode of rarefaction in the range of −115 mm Hg was used at the first stage and −75 mm Hg in the second (in permanent mode). After discontinuing NPWT therapy, local treatment continued using powder sorbents. Bacteriological control of microbial colonization in the wound was performed periodically. Preliminary discussion and signed informed consent preceded the inclusion of patients in the study. Methods of laboratory-instrumental research and treatment protocols were approved by the ethics committee of the Institute of General and Urgent Surgery named after Zaytsev V. T. of the National Academy of Medical Sciences of Ukraine.

## RESULTS

Among the patients treated in the first stage, three observations required two sessions of NPWT, and the other two required three sessions (it depended on the area and depth of the wounds). The second stage of treatment lasted from 5 to 7 days (on an outpatient basis). Following the completion of local treatment, all cases demonstrated a marked synchronization of granulation maturation and marginal epithelialization, leading to wound healing through wound contraction with secondary tension.

**Figure 1:**
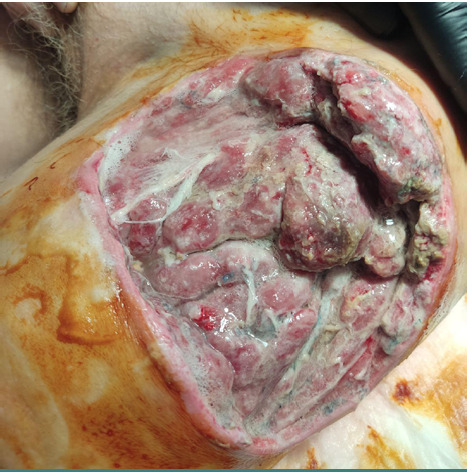
Wound view on admission to the clinic in a 66-year-old male

An illustrative case is a 66-year-old male patient transferred from a military hospital with a shrapnel wound in the upper third of the left thigh, which occurred against the background of type 2 diabetes. Before admission, the wound was treated locally with polyethylene oxide-based ointments (polyethylene glycol). At the time of the admission, the thigh wound was large and deep, measuring 22.0*10.5*4.7 cm, with significant perifocal edema and a fibrin-covered wound bed. The bandage was abundantly soaked with cloudy serous secretions (Figure 1). Microbiological analysis revealed a microbial association: *Proteus mirabilis*: 1.22*10^9^ CFU/ml; *Klebsiella pneumonia*: 1.11*10^5^ CFU/ml; *Enterococcus faecalis*: 1.14*108 CFU/ml.

**Figure 2:**
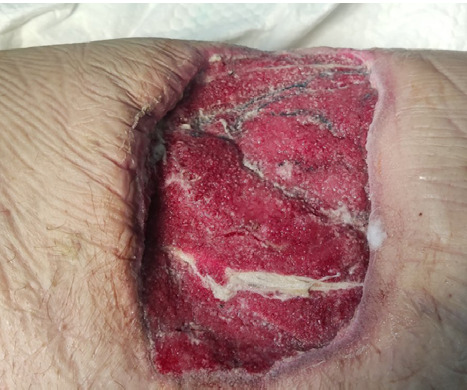
Wound view on the 2^nd^ day after VAC therapy in a 66-year-old male

**Figure 3:**
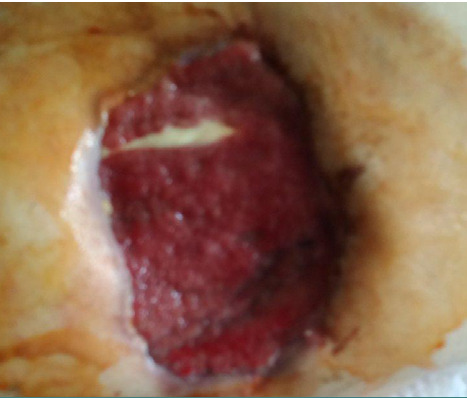
Wound view on the 3^rd^ day after VAC therapy in a 66-year-old male

The decision was made to employ NPWT at a pressure of -115 mm Hg with an intermittent regimen, changing the sorption sponge every two days. The patient tolerated the procedure satisfactorily. The pain decreased sharply, and there was no need to take painkillers; the volume of exudate removed from the wound in two days was 440 ml. After removing the vacuum dressing after two days, the wound defect was 21.0*7.8*3.3 cm with scant exudation and the appearance of granulations. Two days later, the VAC system was re-installed in the same mode. The volume of discharge from the wound was small, and after removing the vacuum system, the wound defect was 19.0*6.8*1.3 cm. The subsequent microbiological analysis detected *Proteus mirabilis*: 10^3^ CFU/ml and *Enterococcus faecalis*: 10^2^ CFU/ml. (Figures 2, 3).

**Figure 4:**
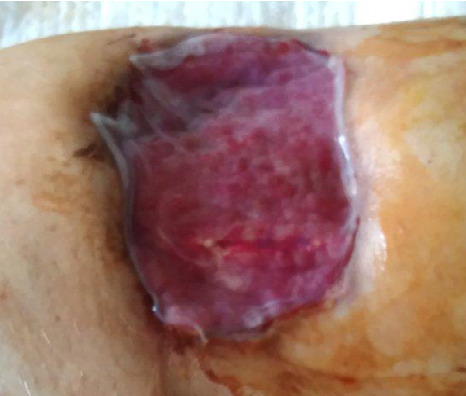
Application of amniotic membrane on the wound of a 66-year-old male

Two days later, the second stage of the treatment was started: the wound surface was covered with a human amniotic membrane (Figure 4), and the VAC system was re-mounted at a vacuum level of −75 mm Hg and used at a constant mode for 7 days.

**Figure 5:**
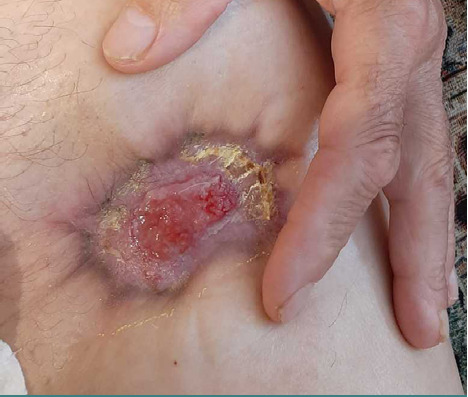
Wound progression on the 17th day of treatment in a 66-year-old male

On the 17^th^ day of treatment, NPWT was discontinued, and further treatment was carried out using alginate dressings on an outpatient basis. On the 30^th^ day of treatment, the patient was examined in a hospital: a superficial wound of 5.0*1.5 cm was detected (Figure 5).

## DISCUSSION

The reparative process of wound healing involves a series of interconnected mechanisms, including hemostasis, inflammation, proliferation, and remodeling, regulated by various cytokines. Wound healing is a dynamic process initiated immediately upon damage and concludes with tissue restoration. Although the restorative processes follow a specific sequence, they can occur simultaneously and overlap in time [6, 8, 11, 26]. This is particularly evident in chronic wounds associated with diabetes, where the pathophysiology is complex and diverse. However, they share a common characteristic of prolonged inflammation, which hinders healing and leads to extensive tissue damage.

Treatment of chronic wounds in diabetes is an extremely complex clinical problem and requires the elimination of damaging factors and the improvement of regional blood circulation, both arterial and venous. If damaging factors are not corrected, the treatment of chronic wounds becomes long-term, with a high risk of relapse. Treatment of chronic wounds should be as atraumatic as possible [26]. There are various approaches to treating chronic wounds, such as the theory of wound bed preparation formulated by Falanga between 2000 and 2002 [27, 28]. Wound bed preparation is a therapeutic strategy used to convert a chronic wound into an acute one and remove both the necrotic component consisting of necrotic tissue and phenotypically altered cells of the edge and base of the wound and the exudate produced by them. The strategy underwent several revisions and refinements, but in general, it is still relevant today, as is the principle of wound healing in a moist environment [29] and the TIME system: T (Tissue) – removal of non-viable, including necrotized tissues; I (Infection) – inhibition of infection; M (Moisture) – control of the level of moisture (wound exudation); E (Edge) – stimulation of reparative processes and/or epithelization [30].

The NPT method was appropriate and effective due to several factors, such as the active removal of excess wound exudates and acceleration of wound tissue deactivation. At the same time, it maintained the balance of moisture in the wound, which is crucial for the healing process, and it stimulated neoangiogenesis. NPT also demonstrated various mediated effects, including eliminating edema, local interstitial formations, strengthening of local blood circulation, convergence of the wound bed, and stimulation of proliferation. There was a decrease in the area and volume of the wound defect, a decrease in the number of hospital-acquired wound infections, an increase in tissue oxygenation, and an increase in the effect of general drug therapy. Furthermore, the use of NPT had socially significant effects, such as reducing the duration and volume of treatment, leading to decreased overall costs and shorter hospitalization periods.

It is possible to consistently apply vacuum-assisted and modern dressings, for example, polymer coatings that ensure gas exchange, preservation of a moist environment, and an optimal temperature regime, which contribute to the maturation of granulation tissue and activation of cell proliferation.

Our study showed that decontamination of the wound below the critical level in the first stage with vacuum therapy was achieved by the 4-5th day compared to the 11th day with traditional methods of local wound treatment.

Applying local negative pressure to the wound bed induces tissue stretching and deformation, which, in turn, significantly influences the cells within the treated tissues. In vitro studies have demonstrated that cell proliferation is enhanced when cells are subjected to stretching under the influence of negative pressure. In contrast, cells not subjected to stretching do not exhibit a similar level of division intensity.

The study investigated this effect and proposed an explanation based on the cytoskeleton structure and indirect connections between the cell wall and nucleus. These connections, observed in the experiment, transmitted mechanical tensile forces from the cell wall to the nucleus, resulting in a cascade of effects characterized by gene expression, increased production of growth factors, and enhanced synthesis of tissue proteins. Subsequently, these changes in cell adaptation led to increased proliferative processes.

Components of placental amniotic membranes included, among other things, the extracellular matrix, the cells themselves, and a complex of regulatory cytokines that promoted tissue growth and modulated inflammation. Placental amniotic membranes have been recognized for their ability to modulate and potentially enhance cell proliferation through the increased secretion of cytokines by various cell types. The amniotic membrane is rich in growth factors, with approximately 226 growth factors identified, including cytokines, chemokines, and growth factors. Additionally, the amniotic membrane contains regulatory inhibitors of metalloproteinases, such as TGF-α, TGF-β, PDGF-AA, PDGF-BB, EGF, bFGF, VEGF, IL-4, IL-10, TIMP-1, TIMP-2, TIMP-4, as well as placental growth factor (PlGF). The placental growth factor plays an important regulatory role in fetal development and pregnancy.

Placental tissue generally does not cause immune rejection because it contains low levels of HLA antigens. In this way, the placental tissues can be cleaned of blood and hazardous materials while preserving the natural biological activity of the tissue for transplantation without complete decellularization. These products in vitro enhanced the synthesis of growth factors and angiogenic factors by skin fibroblasts and significantly increased the number of blood vessels and the number of CD31+ found at the healing sites in mouse models. When performing enzyme-linked immunosorbent assays, it was found that significant amounts of tissue inhibitors of metalloproteinases and interleukins, which were contained in amniotic membrane products, contributed to the reduction of inflammation. Cryopreserved amniotic products have been recognized for their ability to preserve native amniotic stem cells and contain important growth factors and cytokines. In vitro studies have shown that these products enhance the synthesis of adipose tissue stem cells, hematopoietic stem cells, and mesenchymal stem cells during cultivation while attracting stem cells to sites of active regeneration. Animal models have demonstrated an increase in the number of CD34+ cells and hematopoietic stem cells in subcutaneous tissue samples compared to control models. Furthermore, in vitro studies have revealed that amniotic membrane products have anti-inflammatory properties, reducing the inflammatory response and proliferation of alloreactive T cells. Lymphocytes cultured with amnion have shown a decrease in the synthesis of Th1 and Th2 cytokines. Reduced manifestations of alloreactivity, believed to be related to HLA-A, -B, -C, -DR, or beta-2-microglobulin antigens, were determined by the absence of these antigens in the amniotic membrane. This suggests that the immunogenicity of amniotic membrane products may be advantageous compared to bioengineered skin substitutes. Studies evaluating the described properties associated with amniotic tissue have mostly been limited to in vitro experiments and animal models but provide evidence to support a regenerative effect associated with amniotic tissue products.

Applying a vacuum dressing to a transplanted human amniotic membrane improved the adaptation of the flap to the wound surface, made it possible to remove excess wound exudate, and stimulated angiogenesis and reparative properties of the membrane [31, 32]. To apply the vacuum therapy method after covering the wound surface with an amniotic membrane, a negative pressure level of −50 to −75 mm Hg was used with an additional non-adhesive barrier (or dressing) between the absorbent sponge and the membrane. The total duration of such a treatment session was 4 days.

Our study showed that treatment of a diabetic chronic wound using a combination of amniotic membrane and NPWT therapy leads to successful wound healing. On the one hand, the use of NPWT leads to temporary hypoperfusion at the edges of the defect, resulting in the increased local concentration of hypoxia-inducible factor 1α and vascular endothelial growth factor, which enhances neoangiogenesis. In addition, the strengthening of local blood flow helps improve the supply of tissues in the area of the defect with oxygen and nutrients while simultaneously removing the products of tissue viability. Reducing tissue exudation improves healing by reducing the severity of interstitial edema, which contributes to the emergence of chronic defects due to the local compression of cells and tissues. Decreasing the bacterial load on a wound is a critical factor contributing to accelerated healing and is important in achieving final wound closure [33]. Additionally, the utilization of human amniotic membrane as a covering material enhances the formation of the basement membrane, which improves the protective capabilities of the wound against infectious agents. The basement membrane acts as a protective barrier, even in the absence of a complete overlying epithelial layer. A study by Loffelbein *et al*. demonstrated that wound treatment with an amniotic membrane resulted in fewer hypertrophic scars than wounds without an amniotic membrane [34]. Furthermore, Loffelbein *et al*.'s study investigated the use of amniotic membranes in both pig and human models. In the pig model, applying an amniotic membrane prevented scarring and promoted enhanced basement membrane formation [35].

Our study had several limitations. Firstly, the sample size was small, consisting of a limited number of patients with long-term non-healing wounds. Secondly, all patients analyzed were previously treated in other hospitals where they received appropriate therapy, including antibiotics. Thirdly, we cannot judge the quality of their previous treatment only based on a retrospective analysis of their medical records, nor can we accurately indicate whether they considered the risk factors for reducing the reparative processes in wounds in their previous treatment. Future research should consider these factors to provide a more comprehensive and objective assessment of the proposed treatment technology for this specific patient population.

## CONCLUSION

The presented data suggested that NPWT, in combination with biotherapy, was a safe and effective treatment for diabetic wounds, promoted faster wound healing, reduced the need for autodermoplasty, and possibly reduced the need for high amputations.
